# Implementation of a Framework for an Abridged Review Using Good Reliance Practices: Optimising the Medicine Regulatory Review Process in South Africa

**DOI:** 10.1007/s43441-020-00144-0

**Published:** 2020-03-25

**Authors:** Andrea Keyter, Sam Salek, Neil McAuslane, Shabir Banoo, Samvel Azatyan, Stuart Walker

**Affiliations:** 1 Department of Clinical and Pharmaceutical Sciences, School of Life and Medical Sciences, University of Hertfordshire, Hatfield, UK; 2 South African Health Products Regulatory Authority, Pretoria, South Africa; 3 Centre for Innovation in Regulatory Science, 160 Blackfriars Road, London, SE1 8EZ UK; 4 Right to Care, Johannesburg, South Africa; 5 Faculty of Health Sciences, University of the Witwatersrand, Johannesburg, South Africa; 6 World Health Organization, Geneva, Switzerland

**Keywords:** Good reliance practices, Abridged review, Reliance on reference agencies, Regulatory convergence

## Abstract

**Background:**

This study sought to identify criteria and current practices for implementing an abridged review process and understanding barriers and enablers in utilizing reliance models and to offer recommendations for the implementation of an abridged review process in South Africa based on good reliance practices (GRelP).

**Methods:**

A questionnaire was completed by six national regulatory authorities (NRAs) to determine criteria and current practices for implementing an abridged review process. In addition, two focus group discussions were conducted on the practical implementation of an abridged review process based on GRelP.

**Results:**

Participating NRAs indicated that reliance would be placed on one reference agency. Applications submitted to NRAs for an abridged review had to be identical to those submitted to the reference agency. Unredacted reference agency assessment reports would be required to facilitate the abridged review process. A full technical dossier would also be required, but only parts would be assessed during the abridged review. Focus groups indicated that abridged review elements had been identified and should be considered in implementing GRelP.

**Conclusions:**

NRAs strive to improve regulatory performance and accelerate approval times; however, many continue to face challenges due to resource constraints. Increasing workloads, advancing technologies, and limited expertise require NRAs to leverage regulatory convergence initiatives, collaborative registration procedures, and functional regional, continental and international networks to fulfil regulatory mandates. Recommendations for the implementation of an abridged review process and a framework for GRelP have been made with a view to optimise regulatory review processes in South Africa.

## Introduction

National Regulatory Authorities (NRAs) are responsible for ensuring the safety, quality, and efficacy of medicines [1], although access to essential medicines is limited in many low-to-middle-income countries [2]. Disparities in the regulatory capacity of NRAs between low- and high-income countries and the lack of collaboration and work sharing in medicines’ regulation between NRAs have been previously identified [2]. Approximately, 30% of NRAs do not have the necessary capacity in terms of expertise, quality management systems, and human and financial resources to fulfil core regulatory functions [2]. The World Health Organization (WHO) has initiated the development of guidelines for good regulatory practices (GRP) to support NRA efforts to achieve increased efficiency in regulatory systems, higher quality regulation, improved decision-making, and better public health outcomes [2, 3].

The review of the quality, efficacy, and safety of medicines is considered to be one of the key functions of NRAs [4] and the timely review of applications for registration of new active substances (NASs) can significantly improve patients’ access to medicines and consequently impact public health [1]. The implementation of good review practices (GRevP) supports improved regulatory performance and contributes to the advancement of convergence of NRA regulatory requirements [5, 6].This coupled with the alignment of the International Council for Harmonisation of Technical Requirements for Pharmaceuticals for Human Use *(*ICH*)* technical guidelines would create opportunities for reliance based on the regulatory decisions of other NRAs and supports possibilities for work sharing and joint regulatory initiatives [7].

The WHO has defined reliance as “the act whereby an NRA in one jurisdiction may take into account or give significant weight to work performed by another regulator or other trusted institution in reaching its own decision” [8]. The NRAs in resource-limited settings may apply facilitated regulatory pathways (FRPs) to meet patients’ expectations of timely access to medicines and accelerate the regulatory review process by condensing the elements considered in the review of new medicines. Such NRAs remain responsible for the regulatory decisions made through FRPs and in this way are able to maintain sovereignty in making regulatory decisions [8]. The application of FRPs should be developed on appropriate legal frameworks and within the bounds of commensurate resources.

The WHO has developed draft guidance for good reliance practices (GRelP). These GRelP are derived from GRP and fit within the remit of best practices for the regulation of medical products as prescribed by the WHO [2]. GRelP may be implemented across all regulatory processes and applied to all medicines throughout the whole product life cycle, while contributing to an improved healthcare environment through the promotion of fully functional national regulatory systems. Furthermore, NRAs may apply GRelP to advance good governance, transparency, and regulatory convergence, which in turn support good-quality decisions by NRAs and present opportunities for leveraging the regulatory effort of other NRAs while promoting the conservation of limited regulatory resources [2].

This study aimed to provide recommendations for the implementation of an abridged review process and a framework for good reliance practices in South Africa. This work is the first to be carried out in determining the current practices of NRAs in performing an abridged review of a NAS while considering the practicality of the implementation of GRelP.

## Study Objectives

The main objectives of this study were to:

- identify the criteria and current practices within a number of NRAs for implementing an abridged review process;
- conduct focus groups on the practical implementation of an abridged review process for new medicines in the light of the WHO GRelP; and
- develop recommendations for the implementation of an abridged review process based on GRelP in South Africa.

## Methods

### Questionnaire: Criteria and Current Practices for Implementing an Abridged Review Process

A questionnaire, the Abridged Review Process Profile (ARPP), was developed by the Centre for Innovation in Regulatory Science (CIRS) [9] to identify the criteria and current practices that were applied by NRAs for implementing an abridged review process. A number of NRAs have already implemented processes to facilitate an abridged review. The ARPP was distributed to each of the regulatory authorities recruited into the study in Australia, Brazil, Canada, Indonesia, Israel, Thailand, Saudi Arabia, and Singapore as well as the Gulf Health Council.

The ARPP consists of five parts:

Part I: NRA information*—*This part of the questionnaire described the mandate and scope of the NRA as well as its size and type, including information on the number of reviewers within the NRA and their areas of expertise.

Part II: Criteria for product inclusion and reliance on reference agency—The specific criteria applied to determine which products were eligible for inclusion in the abridged review process were recorded. The criteria for the selection as well as how many reference agencies on which to rely were also described.

Part III: Data requirements—This part of the ARPP lists the data requirements for the abridged review. The type of assessment report from the reference agency that would be used to facilitate the abridged review and the level of detail of information that would be required were described.

Part IV: Clinical factors—The clinical factors considered in the benefit–risk evaluation were recorded.

Part V: Enablers and barriers—The perceived enablers and barriers to the implementation of an abridged review were also listed.

### Focus Group: Practical Implementation of an Abridged Review Process for New Medicines and GRelP

Two focus group sessions were conducted with representatives from regulatory authorities, industry, academia, and patient groups. The focus group sessions held in South Africa and Singapore consisted of 16 and 13 participants, respectively, a moderator for facilitating the discussion, and a rapporteur who was responsible for consolidating the results and reporting on the outcomes of the discussion. A brief guideline was prepared for the participants of each focus group that described the discussion topic, provided background information and outlined the objectives of the focus group discussion. A list of questions and issues for consideration were developed and made available to each of the focus groups to further stimulate the discussion.

The first focus group was held in South Africa in March 2018 where the topic of discussion was “The practical implementation of an abridged review process for new medicines: where should an agency focus and what are the practical steps needed to change process and mind-sets?” The second focus group was held in Singapore in March 2019 where the topic of discussion was “The draft Good Reliance Practice Guideline—how practical is it? A stakeholder’s review and discussion.”

In line with efforts to redesign its regulatory approaches and processes, the South African Health Products Regulatory Authority (SAHPRA) initiated an abridged review process in July 2019 in an effort to reduce the evaluation time, which was previously around six years. In addition, it introduced new guidelines together with a Summary of Critical Regulatory Elements (SCoRE) document, which is required to be submitted to SAHPRA with all new applications for registration. These documents were examined in the light of the abridged study described to make recommendations regarding an appropriate framework for such reviews in South Africa in line with GRelP.

## Results

For the purpose of clarity, the results are presented in three parts:

- Part I: Criteria and current practices for implementing an abridged review process
- Part II: Outcomes of focus groups
- Part III: Review of the abridged review process initiated in South Africa

### Part I: Criteria and Current Practices for Implementing an Abridged Review Process

Six out of nine of the regulatory authorities recruited into the study completed the ARPP including: Australia, Brazil, Canada, the Gulf Health Council, Israel, and Thailand. In addition, information from the public domain such as documents published by SAHPRA for public comment and the CIRS workshops held in Singapore and South Africa were included.

#### National Regulatory Authority Information

This part of the questionnaire provided insight into the scope, regulatory mandate and size of the participating NRAs. Among the six responding agencies the total number of staff for medicinal products for human use was 731, 1958, 186, 565, 40, and 38 and the number of reviewers for applications for marketing authorization/ product licences was 115, 134, 186, 247, 29, and 17. The scope and regulatory mandate of the agencies is listed in Table 1.

**Table 1.** Scope and Regulatory Mandate of Participating NRAs.

Factor	Number of Agencies
Type of agency	
Autonomous agency, independent from the Health Ministry administration	2
Operates within the administrative structure of the Health Ministry	4
Scope/remit of agency	
Medical products for human use	6
Medicinal products for veterinary use	4
Medical devices and in vitro diagnostics	4
Blood and blood products	1
Main agency activities	
Marketing authorization/product licences	6
Post-market surveillance	4
Laboratory analysis of samples	2
Clinical trial authorization	4
Advertising regulation	4
Price regulation	3
Site inspection	4
Other	1

#### Criteria for Product Inclusion and Reliance on Reference Agency

The participating NRAs concurred that one of the key criteria for product inclusion was the submission of an application for an NAS that was identical to that approved by, or submitted to, the reference agency. The application submitted had to be identical in terms of dosage form, strength, formulation, and manufacture. Three of the participating NRAs reported that the proposed indication for the medicine would need to be based on broadly similar population demographics, disease profiles, and expectations regarding public health outcomes between the NRA and the reference agency. Most of the participating NRAs confirmed that NASs were eligible for inclusion but one NRA stated that the abridged review would only be applicable to biological products, while biosimilars would be excluded. One NRA specified that the NAS in question had to be approved as well as being available on the market in the reference agency country.

The participating NRAs documented inclusion criteria relating to the timeframe between the submission of the NAS application to the reference agency and the submission to the NRA. Two of the NRAs did not impose restrictions in terms of this time frame, while two NRAs indicated that applications that had been submitted to the reference agency more than 2 years before would not be considered, and one NRA indicated that a new guideline had been drafted that echoed this requirement. One NRA stated that a timeframe of not more than one year would be applied for the quicker evaluation route. The participating NRAs indicated utility and compliance to global standards and technical guidelines as well as the availability of reference agency assessment reports, integrity in decision-making, and transparent communication as key considerations in selecting a reference agency. Six of the participating NRAs selected the United States Food and Drug Administration (USFDA) and the European Medicines Agency (EMA) as reference agencies on which reliance would be placed for the purposes of implementing an abridged review. Four of the NRAs indicated that reliance was also placed on the Medicines and Healthcare Products Regulatory Agency (MHRA) of the United Kingdom and the Swiss Agency for Therapeutic Products (Swissmedic) while other reference countries considered for reliance included Australia (3), Canada (3), Japan (3), New Zealand (1), Norway (1), Singapore (1), Iceland (1) and the World Health Organization Prequalification of Medicines Programme. Six of the participating NRAs stated that reliance would be placed on only one reference agency in the application of the abridged review process and one NRA specified two reference agencies, namely the USFDA and the EMA. In the event that reliance was placed on more than one reference agency and a difference in the regulatory decisions of the two reference agencies was noted, the NRA would apply the reference regulatory decision most appropriate to the requirements of the jurisdiction.

#### Data Requirements

##### Assessment Report

Five of the participating NRAs stated that unredacted assessment reports would be required to facilitate the abridged review process. Three of the six NRAs indicated that redacted reports could be used, provided that these reports were only lightly redacted and that all the necessary information was available. Also required is a list of questions to sponsors and their responses as well as post-marketing commitments. Three of the NRAs made use of public assessment reports (PARs) that are available in the public domain. Five of the six NRAs indicated that while only parts of the technical document would be reviewed during an abridged review, it was a requirement that a full ICH/Association of Southeast Asian Nations (ASEAN) common technical document (CTD) had to be submitted for the abridged review. All of the six participating NRAs provided insight into the depth of the CTD review during the abridged review (Table 2).

**Table 2.** Depth of Review of the Common Technical Document by the National Regulatory Authority in the Abridged Review.

Area of the CTD Reviewed	Only Reviewed If There was a Query	Verification for Completeness of Data	Selective Detailed Review	Detailed Review and Assessment Report Prepared
Quality (CMC)	0	0	3	3^a^
Human Pharmacology	3^b^	1	0	2^b^
Clinical	1	1	0	4^c^
Non-clinical	3^b^	1	0	2^b^

*CTD* common technical document; *CMC* chemistry, manufacturing and controls; *NRA* national regulatory authority.

^a^Reflects the current situation, however, in the new draft guidelines the NRA will only review the reference agency assessment report, but may review data in CTD if necessary.

^b^One NRA indicated that currently the level of review was dependent on the product and availability of the reference agency assessment report. The new draft guidelines state that the NRA will only perform a review of the data in the CTD if an issue was identified by the reference agency.

^c^One NRA stated that the new draft guidelines described that only the pivotal studies would be reviewed.

##### Application

In support of the requirement for an abridged review, participating NRAs verified that applications submitted should be identical to that approved by the reference agency. All of the participating NRAs required the dosage form and strength of the NAS to be identical with that of the NAS submitted to the reference agency. All of the six participating NRAs required that the ingredients of the respective NAS be identical and four of the NRAs required that the indications, dose, and warnings and precautions of the NAS be identical. All of the NRAs accepted a closely similar product label to that submitted to the reference agency. During the abridged review process, NRAs may choose to perform a detailed review of the reference agency assessment reports in lieu of performing an internal review of the CTD or review areas of the reference agency assessment report in the event that the reviewer identifies an issue. Five of the participating NRAs indicated that a detailed review of the reference agency assessment report was performed during the abridged review. The areas of the reference agency assessment report relating to quality/chemistry, manufacturing and controls (CMC), human pharmacology, clinical and non-clinical data were reviewed in detail by the NRAs as part of the abridged review.

#### Clinical Factors

The majority of the participating NRAs indicated that clinical factors such as differences in medical practice, national disease patterns, and unmet medical needs were taken into account during the clinical evaluation and the benefit–risk assessment that was conducted during the abridged review. The majority of the NRAs indicated that ethnic factors were also sometimes considered during an abridged review.

#### Enablers and Barriers

In Part V of the questionnaire, the participating NRAs provided insight into the perceived enablers and barriers that impacted on the implementation of an abridged review (Table 3).

**Table 3.** Enablers and Barriers Identified by NRAs in Implementing an Abridged Review.

Enablers	Barriers
Availability of the unredacted reference agency assessment reports	Not receiving the unredacted reference agency assessment reports from the applicant
Availability of the list of questions from the reference agency to the applicant and post-approval commitments	Resistance from applicants to apply for the abridged review process as requirements for supporting documents could not be met
Approval of an NAS within two years of the reference agency	Inadequate transparency with regard to reference agency decision-making process
Applicants who are willing to answer questions throughout the course of the review rather than at the end	Benefit-risk assessment is not sufficiently detailed and presents challenges in application to the local NRA population
Increased communication and interaction with other agencies	Differences or diversity in regulatory requirements between the NRA and the reference agency
Saves resources as the assessment report of the reference agency may be used for the review instead of contracting an external expert to conduct the review	The reliance on work conducted by another agency requires a culture shift to mitigate unease that reliance will result in a loss of local expertise

*NAS* new active substance, *NRA* national regulatory authority.

### Part II: Outcomes of Focus Group Discussions

The outcomes of the first focus group session that was held in South Africa in March 2018 resulted in recommendations for consideration in the practical implementation of an abridged review process for NASs. The participants concluded that the elements constituting an abridged review had to be identified. It was recognised that the requirements for applications submitted for abridged review to the NRAs participating in the discussion, were similar. The participants also agreed that while information such as reference agency assessment reports were available in the public domain, these were often heavily redacted and ill equipped to support regulatory decisions made by NRAs during the abridged review process. The participants endorsed the recommendation to perform a study to identify what components of a dossier are evaluated by NRAs when performing an abridged review.

The outcomes of the second focus group session, which was held in Singapore in March 2019 resulted in recommendations for consideration in the review of the practicality of the draft WHO GRelP guideline. The participants agreed that reliance practices were largely based on the use of information or regulatory decisions of a trusted source/reference agency. It was acknowledged that reliance practices were used in diverse applications and participants commented that shared inspection reports and CMC reports could be used to confirm the quality of an NAS without duplicating regulatory efforts. Participants endorsed the application of a phased approach in the implementation of GRelP and commented positively regarding the requirement to provide a summary of the benefit-risk assessment and findings and/or recommendations prepared by the reference agency. The participants endorsed the outcomes of the study, which identified which NRAs have implemented reliance pathways and what the requirements were for such pathways.

### Part III: Evaluation of the Abridged Review Process Initiated in South Africa

SAHPRA initiated an abridged review process in 2019 in an effort to limit the evaluation time of medicines that have been registered by reference agencies recognised by SAHPRA. All NASs, including biological medicines, generic medicines, type II variations, and major line extensions would be eligible for an abridged review [10]. Similar to the requirements of the participating NRAs in this study, SAHPRA required the submission of an application that was materially the same as that submitted to a reference agency recognised by SAHPRA. The EMA was considered as the default reference agency by SAHPRA for reliance, however, the USFDA, Japanese Pharmaceuticals and Medical Devices Agency (PMDA), Health Canada, Swissmedic, TGA, and MHRA were also listed as recognized agencies. Applicants were required to submit the full CTD and were also requested to submit unredacted assessment reports from reference agencies. Where these were not available, applicants were requested to submit a request to the reference agency to make the relevant unredacted assessment reports available to SAHPRA. SAHPRA also requested the submission of any correspondence between the applicant and the reference agency relating to safety and efficacy or queries, the risk management plan, or benefit–-risk decisions [10]. The Clinical Guideline published by SAHPRA in July 2019 described the requirements for the clinical evaluation of medicines using the abridged review [10]. The guideline indicated that only the overviews of the pre-clinical and clinical data described in CTD modules 2.4 and 2.5 would be reviewed, however, reviewers were at liberty to perform a full review of CTD modules 4 and 5 if it was deemed necessary [10].

The new Clinical Guideline indicated that the summary basis for registration (SBRA) document that was previously required by SAHPRA to support clinical evaluation of a medicine was no longer required and would be replaced by the clinical overviews and summaries and the SCoRE document. The SCoRE document was required to be submitted with all new applications for registration [11] and was required to be submitted as part of CTD module 3.2.R.8 (Other) in addition to the Quality Overall Summary. Applicants were also required to submit the latest periodic safety update report (PSUR)/periodic benefit–risk evaluation report (PBRER) and reference package insert approved by the reference agency. SAHPRA also indicated that two additional reliance pathways had been developed for medicines that had been pre-qualified by the WHO and for medicines that had been reviewed through the Zazibona collaborative review procedure [11].

## Discussion

### Practical Implementation of an Abridged Review Process

Strategies initiated by NRAs to leverage international collaboration in the form of reliance and referencing to enhance regulatory performance have been endorsed by the WHO [2]. The participants in the focus groups agreed that there is a definite need for NRAs to use FRPs such as an abridged review to improve regulatory efficiencies. The abridged review is based on the premise that the review time would be decreased as reliance on the assessment report of a reference agency and placing weight on the regulatory decision of a trusted NRA eliminates the need to perform a full assessment of the quality, safety, and efficacy data provided in the technical dossier. Typically, NRAs rely on the decision of one reference agency in support of an abridged review. Applications submitted to NRAs for an abridged review should be identical to those submitted to the reference agency. An abridged review of a NAS relies on the scientific, evidence-based assessment of the NAS by a reference agency. Subsequently, the NRA may review the reference agency’s assessment report and conduct an abridged review of certain parts of the technical dossier in support of local requirements. “The relevance of the use of the NAS under local conditions or in a local population may be determined by reviewing the quality data in relation to climatic conditions and distribution infrastructure and a benefit–risk assessment in relation to its use in the local ethnic population, medical practice/culture and patterns of disease and nutrition” [12]. Enablers supporting the implementation of an abridged review include the availability of unredacted reference agency assessment reports, increased communication and interaction between NRAs and reference agencies, and continued efforts to ensure that regulatory decisions are based on sound regulatory processes and standards.

### Practical Implementation of Good Reliance Practices

The recommendations from several WHO International Conference for Drug Regulatory Authorities (ICDRA) meetings highlighted that the desired public health goals can only be achieved through collective efforts of regulators and other stakeholders [2]. According to the WHO guideline on GRP, reliance is defined as “the act whereby the NRA takes into account and gives significant weight to—i.e., totally or partially rely upon—evaluations performed by another NRA or trusted institution in reaching its own decision” [3]. As more agencies consider reliance pathways, GRelP, defined as “practices that facilitate the implementation of a systematic and optimised framework by NRAs where reliance approaches can be effectively incorporated and consistently applied to minimise duplication of work” are in the process of being developed [13]. The GRelP have been drafted by the WHO to support the systematic and consistent implementation of reliance frameworks within regulatory systems [2]. Through the introduction of such GRelP, NRAs are able to redirect limited resources to core regulatory functions that can only be performed by the NRA with an aim of accelerating patients’ access to medicines. The implementation of GRelP provides an opportunity for NRAs with limited expertise to rely on the technical assessment of reference agencies for complex medical products and consequently provide a solution for timely registration and access to advanced medicines by the local population [2]. The NRA that has relied on the regulatory assessment of another authority remains responsible and accountable for the decisions taken, and therefore their sovereignty of their decision-making process and outcomes is retained [3, 8].

In its publication “Regulatory reliance principles: concept note and recommendations*”* the Pan American Health Organization (PAHO) specified that current regulatory capacity, the needs of an efficient regulatory system and consideration of how the implementation of reliance models may contribute to enhancing the performance of an NRA should form the basis upon which NRAs decide to adopt reliance models and implement GRelP [14]. The key operational principles of reliance models according to PAHO are sovereignty, transparency, consistency, legal basis, and competency. Understanding these principles should guide and inform decision-making by NRAs contemplating the adoption and implementation of reliance practices [14].

National regulatory authorities can tailor the application of these principles to meet the individual needs of national health and regulatory systems [14]. However, the foundation for the implementation of a reliance model is dependent on the knowledge of or information gained from a trusted source that has based regulatory reviews and decision-making on sound scientific evidence, global standards, and robust regulatory frameworks. In this way, trust between NRAs becomes a critical component of reliance as confidence is built through trustworthy networks [14].

Further initiatives to improve trust amongst NRAs have contributed to the reinforcement of reliance structures [14]. These include the benchmarking of national regulatory systems of WHO Member States, by the WHO, using the standardized WHO global benchmarking tool [15] and the evaluation of NRA inspection capacities by the Pharmaceutical Inspection Co-Operation Scheme (PIC/S) [16].

In a March 2019 presentation, Bee also outlined the principles of GRelP. Like the PAHO publication on reliance principles [14], Bee also listed NRA sovereignty and consistency among these principles; that is, the implementation of GRelP should not undermine the authority of the NRA as underwritten by the relevant legal framework that supports the regulatory mandate [17]. In addition, the convergence of regulatory requirements among NRAs underpins the success of GRelP, which in turn facilitates enhanced decision-making [17]. Furthermore, the reliance models used for regulatory decision-making should be applied consistently and the decision-making process must remain evidence-based and in compliance with GrevP [17]. Finally, reliance models used to support regulatory decision-making should be extended across the product life cycle to support the post-market robustness of the decision with respect to the local population [17].

Regulatory efficiency could be increased through GRelP support which in turn contributes towards regulatory system strengthening. However, NRAs should continue to develop their regulatory capabilities and develop reliance models based on a set of key principles (Table 4) [2].

**Table 4.** Key Principles in the Development of Reliance Models—Adapted with Permission [2].

Outcome Orientation	Efforts Should Lead to Measurable Public Health Gains
Operational flexibility	One approach may not be appropriate for all situations
Pragmatism	Employing a stepwise approach that builds on successes and lessons learned
Utilising best international practices	Importance of common requirements and approaches based on international best practices and standards, such as the Common Technical Document (CTD), in achieving optimal outcomes
Accountability	The work needs to be planned and staffed appropriately and the outputs need to be implemented consistently, predictably, and transparently

Reliance models that may be used to facilitate the review of medicines include mutual recognition, referencing decisions using unredacted assessment reports of reference agencies or WHO prequalification, work sharing such as the European Union decentralised procedure and the Zazibona process in the Southern African Development Community (SADC) region, and joint assessment such as the WHO-East African Community (EAC) joint assessments/inspections and the ASEAN joint assessments [2, 17],

The GRelP system must be integrated into the frameworks developed by NRAs to support the implementation of reliance models. It is, therefore, important that reliance models are built on a legal and regulatory foundation that supports international cooperation and exchange of information with other NRAs [17]. This might initially rely on NRA leverage of existing international collaborative platforms to initiate and expedite the implementation of reliance models [17]. NRAs should ensure that both internal and external stakeholders understand and accept the proposed reliance model [17]. Thus, providing clear guidance to applicants and defining the relevant requirements for eligibility criteria, submission requirements, timelines, and registration pathways is recommended to facilitate the process and ensure the intended outcomes [7]. Furthermore, NRAs should ensure that implementation of reliance models is underpinned by capacity-building strategies and rolled out effectively to support the success of such initiatives while continuing to enhance regulatory competencies to complement reliance models [17].

Reliance models may be used by NRAs to support the initial approval of an NAS as well as the management of post-approval variations. While NRAs may rely on the decisions made by reference agencies, they should remain cognisant of the possibility that certain NASs may be developed in a manner that allows for expedited approval, based on an abbreviated data set supported by well-defined post-approval commitments [7]. Transparent decision-making processes must also be in place to ensure that the basis for the approval or rejection of a NAS is adequately documented.

## Conclusions

While NRAs strive to improve regulatory performance and work toward achieving accelerated approval times for NASs, many NRAs continue to face challenges due to resource constraints. Increasing workloads, advancing technologies and limited expertise create the need for NRAs to leverage regulatory convergence initiatives, collaborative registration procedures and functional continental networks to fulfil their regulatory mandates [2].

The SAHPRA has faced similar challenges and has taken steps towards embracing reliance models and employing an abridged review process for NASs. Key recommendations to ensure the success of the proposed reliance model for an abridged review and the implementation of GRelP by SAHPRA should include:

- Formalising the implementation of GRelP;
- Continuing to place reliance on trusted reference agencies that have met the requirements of standardised regulatory benchmarking tools;
- The verification that the NAS applications submitted to SAHPRA are materially the same as that submitted to a reference agency recognised by SAHPRA;
- Limiting the scope of the abridged review to a:
  o Detailed review of clinical data including consideration of clinical factors such as differences in medical practice, national disease patterns, unmet medical needs and ethnic factors;
  o Review of the quality data and non-clinical data only in the event of query; and
  o Selective review of human pharmacology data.

The implementation of abridged reviews by SAHPRA based on these recommendations of GRelP should have a major impact on regulatory review times which over the last four years (2015–2018) were in excess of five years. Thus, this approach, if continued and endorsed by SAHPRA, will ensure the timely access to new medicines for patients.
